# Transcriptome MicroRNA Profiling of Bovine Mammary Glands Infected with *Staphylococcus aureus*

**DOI:** 10.3390/ijms16034997

**Published:** 2015-03-04

**Authors:** Rui Li, Cheng-Long Zhang, Xiang-Xiang Liao, Dan Chen, Wen-Qiang Wang, Yi-Hui Zhu, Xiao-Han Geng, De-Jun Ji, Yong-Jiang Mao, Yun-Chen Gong, Zhang-Ping Yang

**Affiliations:** 1 College of Animal Science and Technology, Yangzhou University, Yangzhou 225009, China; E-Mails: lirui871025@126.com (R.L.); yangdazhangcl@163.com (C.-L.Z.); kevin89free@163.com (X.-X.L.); dan0513_2015@126.com (D.C.); 15371254521@163.com (W.-Q.W.); zhuyh2015@163.com (Y.-H.Z.); xhgeng2012@163.com (X.-H.G.); djji@yzu.edu.cn (D.-J.J.); cattle@yzu.edu.cn (Y.-J.M.); 2 The Centre for the Analysis of Genome Evolution and Function (CAGEF), University of Toronto, Toronto, ON M5S 2J7, Canada; E-Mail: yunchen.gong@utoronto.ca

**Keywords:** microRNAs, mastitis, *Staphylococcus aureus*, Solexa sequencing, qRT-PCR

## Abstract

MicroRNAs are small non-coding RNA molecules that are important regulators of gene expression at the post-transcriptional level. miRNAs impact the processes of cell proliferation, differentiation and apoptosis. Thus, the regulation of miRNA expression profiles associated with mastitis will be conducive for its control. In this study, *Staphylococcus aureus* (*S. aureus*) was administered to the mammary gland of Chinese Holstein cows to construct a bacteria-type mastitis model. Total RNA was isolated from bovine mammary gland tissue samples from the *S. aureus*-induced mastitis group and controls. miRNAs were analyzed using Solexa sequencing and bioinformatics processing for the experimental group and control group. Two miRNA libraries were constructed respectively. A total of 370 known bovine miRNAs and 341 novel mi RNAs were detected for the *S. aureus* and 358 known bovine miRNAs and 232 novel miRNAs for control groups. A total of 77 miRNAs in the *S. aureus* group showed significant differences compared to the control group. GO (Gene Ontology) analysis showed these target genes were involved in the regulation of cells, binding, *etc.*, while KEGG (Kyoto Encyclopedia of Genes and Genomes) analysis showed that these genes were enriched in endocytosis, and olfactory transduction pathways involved in cancer. These results provide an experimental basis to reveal the cause and regulatory mechanism of mastitis and also suggest the potential of miRNAs to serve as biomarkers for the diagnosis of mastitis in dairy cows.

## 1. Introduction

Bovine mastitis is an inflammatory reaction caused by the persistence of pathogens (e.g., *Staphylococcus aureus* and *Streptococcus*) within the mammary glands. It has long been considered to be one of the most economically important diseases in the dairy industry [1]. Efforts at control of mastitis include studying the mechanisms of host responses towards infecting pathogens and the development of appropriate control strategies. Transcriptome studies of bovine mammary epithelial cells challenged *in vitro* with mastitis pathogens revealed very different mechanisms of host innate immune responses to pathogens [2,3,4,5]. Understanding the regulation of the immune response at the molecular level is particularly important for the control of mastitis pathogens [6]. MicroRNAs (miRNA) are small non-coding RNA molecules that are approximately 18–25 nucleotides (nt) in length and are produced by the organism’s own genomic transcription. One of the main functions of miRNA is to regulate the basic aspects of the processes of life, such as cell growth, proliferation, differentiation and apoptosis, aging, and death [7,8]. The role of miRNA in the regulation of immune responses has been well reviewed [9,10].

It also has been suggested that miRNA may modulate epithelial immune responses during every step of the innate immune network [11]. MicroRNAs have also been clearly demonstrated to have important roles in regulating responses to infection [12]. It was shown that miR-155 promotes the production of TNF-α in human embryonic kidney cells (HEK-293), indicating the positive role of miR-155 to modulate the release of inflammatory mediators [13]. However, the roles that miRNAs play in regulating immune response to infection is less understood in dairy cow. Several studies have examined the miRNA expression changes in response to bacterial infections associated with bovine mastitis. For example, five inflammation-related miRNAs (miR-9, miR-125b, miR-155, miR-146a and miR-223) were differentially expressed in bovine CD14+ monocytes stimulated with either LPS or *Staphylococcus aureus* enterotoxin B (SEB) [14].The expression of 14 miRNAs (miR-10a, -15b, -16a, -17, -21, -31, -145, -146a, -146b, -155, -181a, -205, -221, and -223) associated with regulation of innate immunity and mammary epithelial cell function in tissue challenged with *Streptococcus uberis*, Three miRNAs (181a, 16, and 31) were downregulated approximately 3- to 5-fold and miR-223 was upregulated approximately 2.5-fold in infected *versus* healthy tissue [15]. Increasingly, more studies have employed high-throughput sequencing to profile changes in miRNA expression in response to challenge with bovine mastitis-causing pathogens. Twenty-one miRNAs were identified as differentially expressed in bovine mammary epithelial cells challenged *in vitro* with live *S. uberis* [16].

Most of these studies were focused on research at the *in vitro* cell level, while few studies were *in vivo*. Furthermore, the mechanism by which miRNAs modulate this robust immune response in bovine mammary epithelial cells in the presence of the *S. aureus* bacteria is poorly understood.

To investigate the role of miRNA in host defenses against *S. aureus*, we artificially administered *S. aureus* to the mammary glands of Chinese Holstein cows to construct a bovine mastitis model. miRNA properties were compared between the *S. aureus* infected group and the control group. Finally, we discussed possible functions of the identified miRNAs. These findings provide an experimental basis for investigating the pathogenesis of *S. aureus*-type mastitis.

## 2. Results

### 2.1. Establishment of Mastitis Model

**Figure 1:**
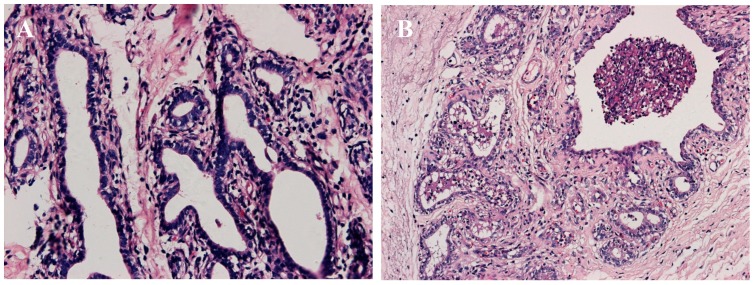
HE staining of mammary glands tissue (400×). (**A**) Control group; (**B**) *S. aureus* group.

Twenty-four hours following the administration of the *S. aureus*, swelling, pain, warmth and redness was present in the cow breast. Flocculent precipitate appeared in milk and milk somatic cell count (SCC) was significantly increased (>2,000,000 cells/mL). All these symptoms indicated the mastitis model was successfully established. In addition, HE staining was employed to verify the mastitis model. As showed in Figure 1, the integrity of the breast structure in the control group evident; the mesenchyme was thin and uniform, without inflammation and hyperplasia phenomenon; the breast acini were tightly packed, with neatly arranged monolayer mammary epithelial cells, showing no pathological changes. In the *S. aureus* group, however, obvious lesions appeared in breast, the atrophied acinar cavity shrank, and the mammary epithelial cells were not closely connected. A lot of desquamated mammary epithelial cells, polymorphonuclear neutrophils, macrophages, lymphocytes and other inflammatory cells were observed in the acinar cavity.

### 2.2. Standard Bioinformatics Analyses

**Figure 2:**
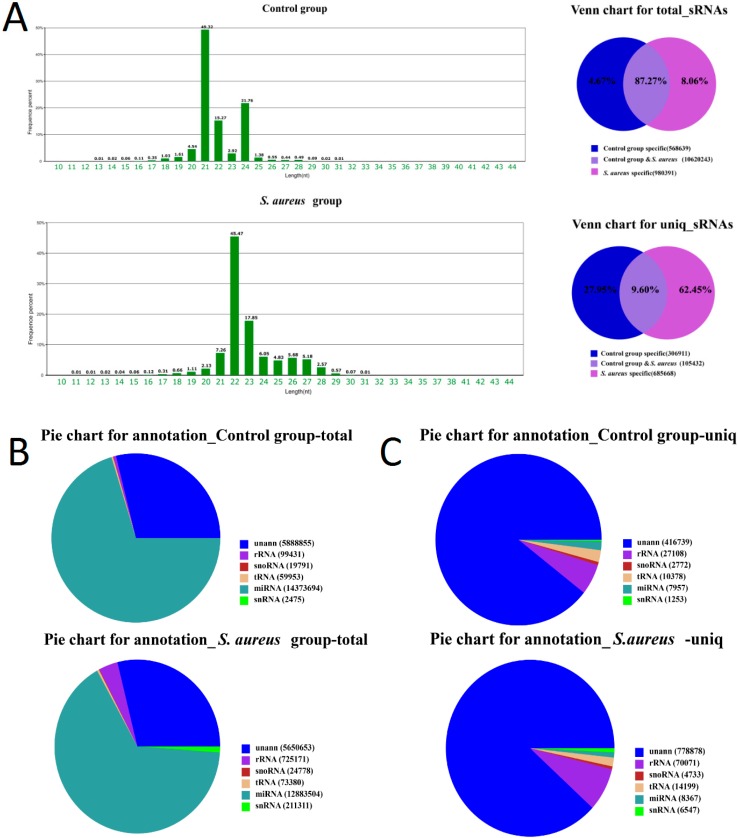
(**A**) Length distribution of two groups of small RNAs; (**B**) Summary of the common and specific tags of two samples; (**C**) Distribution of small RNA among different categories in the control group and *S. aureus* group.

After removing low-quality reads and adaptor sequences, 20.85 million clean reads and 18.50 million clean reads were obtained from the normal and *S. aureus* libraries, respectively, and were used to summarize the length distribution. The results revealed that most clean reads were 20–24 nt in length, which is consistent with the typical size range of small RNAs generated by Dicer. The results indicated that the clean reads included a large number of miRNA sequences (Figure 2A).

The sum of small RNA types was 1,098,011, with a total number of 12,169,273 small RNAs. Two types of small RNAs with 105,432 reads were detected, accounting for 9.6% of the total; additionally, 10,620,243 common RNAs were detected, accounting for 87.27% of the total (Table S1).

A total of 306,911 small RNAs were unique to the healthy control group, accounting for 27.95% of the total; a total of 568,639 sRNAs were unique, accounting for 4.67% of the total. The number of sRNAs unique to the *S. aureus-*treated group was 685,668, accounting for 62.45% of the total; a total of 980,391 unique small RNA accounted for 8.06% of the total (Figure 2B).

### 2.3. Characteristics of Known miRNAs

The use of alignments of small RNA to miRNA precursors of corresponding types in miRBase resulted in the identification of 358 miRNAs, 53 miRNA-5p, 51 miRNA-3p, and 488 miRNA precursors in the control group. The small RNA types matched miRNA precursors with 8066 unique tags and a total of 14,380,650 reads (Table S2). In the *S. aureus* group, 370 miRNA, 55 miRNA-5p, 53 miRNA-3p, and 511 miRNA precursors were identified. The small RNA types matched miRNA precursors with 8539 unique tags and a total of 12,890,726 reads.

All sRNAs were compared within categories. No representation by unannotated sRNAs was found for any sRNA category. The distributions of small RNAs in each group are listed in Tables S3 and S4.

The majority of sRNAs identified in the control group corresponded to miRNAs (70.31%). However, the miRNA types (unique sRNAs) accounted for a smaller proportion (1.71%) containing mostly unannotated small RNA (89.39%). Total miRNAs in the *S. aureus* group accounted for 65.84% of total sRNAs. miRNA types (unique sRNAs) accounted for a small proportion (0.95%), while unannotated small RNA accounted for 88.23% of total sRNAs (Figure 2C).

### 2.4. Novel miRNA Predictions

miRNA hairpins are mainly located in intergenic regions, introns or reverse repeats in coding sequences. The characteristic hairpin structure of miRNA precursors can be used to predict novel miRNAs. We used the Mireap prediction software (available online: http://sourceforge.net/projects/mireap/) to predict novel miRNA by exploring secondary structure, the Dicer cleavage site and minimum free energy of the unannotated small RNA tags that could be mapped to the genome. The prediction was performed based on the conditions described in the Mireap instructions. In total, we identified 232 novel miRNAs in the control group and 341 novel miRNA in the *S. aureus* group.

### 2.5. Differential Expression of miRNAs

**Figure 3:**
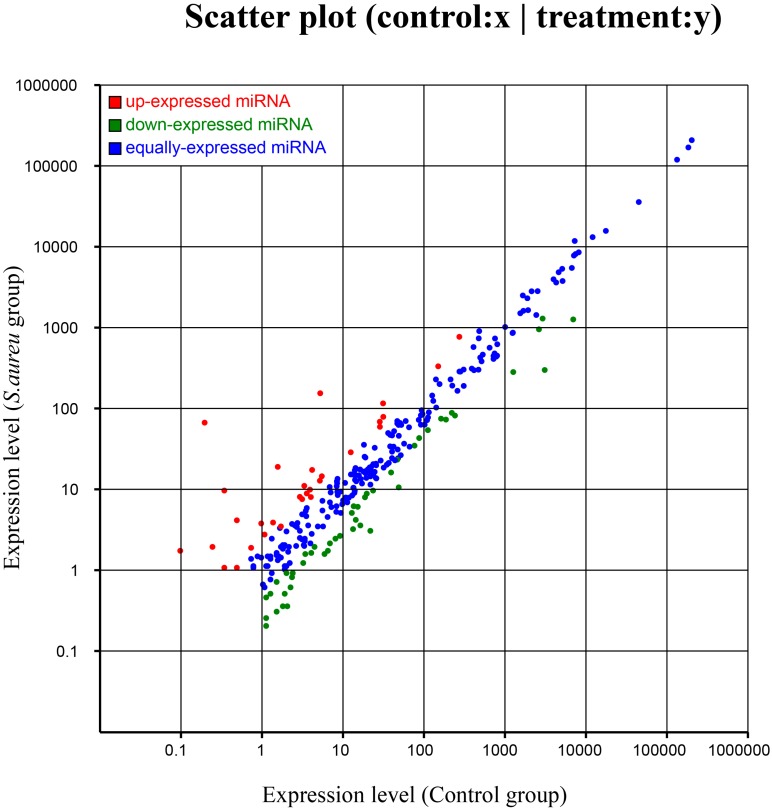
Differential expressions of known miRNAs in MG (Mammary Gland) between the control group and *S. aureus* group (red points represent miRNAs with fold change >2, blue points represent miRNAs with 0.5 < fold change ≤ 2, and green points represent miRNAs with fold change ≤0.5).

Known miRNAs were compared between the two groups to identify differentially expressed miRNA. A total of 77 differentially expressed miRNA were identified between the *S. aureus*-treated and control groups (Table 1). Differences in miRNA expression between the two groups are illustrated by plotting log2−ratios to create Scatter Plot figures (Figure 3).

**Table 1:** Differential expression of known miRNAs in bovine mammary glands between the *S. aureus* and control groups. * *p* < 0.05, ** *p* < 0.001.

miR-Name	A1B1C1-std	A2B3C4-std	Fold-Change (log2 A2B3C4/A1B1C1)	*p*-Value	Expression Level	Significance Level
bta-miR-1246	0.1957	66.79	8.4148	0.00	up	**
bta-miR-223	5.2338	154.28	4.8815	0.00	up	**
bta-miR-184	0.3424	9.6582	4.818	3.68 × 10^−49^	up	**
bta-miR-2313-3p	0.0978	1.7375	4.151	4.92 × 10^−9^	up	**
bta-miR-1298	1.5652	18.959	3.5984	7.68 × 10^−78^	up	**
bta-miR-2887	0.4891	4.1392	3.0812	6.65 × 10^−16^	up	**
bta-miR-1940	0.2446	1.9419	2.989	6.57 × 10^−8^	up	**
bta-miR-877	4.1577	17.375	2.0631	7.00 × 10^−40^	up	**
bta-miR-2484	0.9783	3.7815	1.9506	3.37 × 10^−9^	up	**
bta-miR-451	31.256	115.64	1.8875	3.51 × 10^−227^	up	**
bta-miR-21-3p	3.3261	11.038	1.7306	9.25 × 10^−21^	up	**
bta-miR-2422	0.3424	1.0731	1.648	5.72 × 10^−3^	up	**
bta-miR-3141	1.3696	3.8837	1.5037	6.05 × 10^−7^	up	**
bta-miR-142-5p	273.33	769.54	1.4934	0.00	up	**
bta-miR-142-3p	2.9348	8.0741	1.46	1.69 × 10^−12^	up	**
bta-miR-339b	5.4783	14.513	1.4055	3.42 × 10^−20^	up	**
bta-miR-2468	0.7337	1.8908	1.3657	1.28 × 10^−3^	up	**
bta-miR-138	1.0761	2.7595	1.3586	1.01 × 10^−4^	up	**
bta-miR-2904	3.9131	9.9137	1.3411	2.21 × 10^−13^	up	**
bta-miR-486	31.452	78.799	1.325	1.29 × 10^−93^	up	**
bta-miR-130b	3.5707	8.8917	1.3163	7.60 × 10^−12^	up	**
bta-miR-2284w	5.1848	12.775	1.301	4.04 × 10^−16^	up	**
bta-miR-425-3p	3.1305	7.5631	1.2726	7.85 × 10^−10^	up	**
bta-miR-2284ab	28.468	68.476	1.2663	3.65 × 10^−76^	up	**
bta-miR-2284z	12.473	28.719	1.2032	1.58 × 10^−30^	up	**
bta-miR-132	149.92	332.26	1.1481	3.10 × 10^−310^	up	**
bta-miR-2332	0.4891	1.0731	1.1336	3.73 × 10^−2^	up	*
bta-miR-6529	28.566	59.329	1.0545	7.46 × 10^−50^	up	**
bta-miR-935	1.7120	3.4749	1.0213	4.95 × 10^−4^	up	**
bta-miR-2284aa	4.0109	8.023	1.0002	1.82 × 10^−7^	up	**
bta-miR-99a-3p	46.859	23.302	−1.0079	7.89 × 10^−37^	down	**
bta-miR-365-3p	87.262	43.079	−1.0184	3.29 × 10^−68^	down	**
bta-miR-200b	111.72	53.964	−1.0498	3.77 × 10^−91^	down	**
bta-miR-455-5p	1.5163	0.7154	−1.0837	1.71 × 10^−2^	down	*
bta-miR-1249	3.424	1.5842	−1.1119	2.25 × 10^−4^	down	**
bta-miR-139	162.54	74.762	−1.1204	3.41 × 10^−146^	down	**
bta-miR-2431-3p	2.0055	0.9198	−1.1246	4.58 × 10^−3^	down	**
bta-miR-133a	13.549	6.1833	−1.1317	7.30 × 10^−14^	down	**
bta-miR-196a	76.452	34.698	−1.1397	1.19 × 10^−71^	down	**
bta-miR-664b	19.663	8.8406	−1.1533	5.39 × 10^−20^	down	**
bta-miR-1	2917.8	1294.4	−1.1726	0.00	down	**
bta-miR-874	4.4511	1.9419	−1.1967	7.91 × 10^−6^	down	**
bta-miR-503-5p	18.685	7.9719	−1.2289	6.26 × 10^−21^	down	**
bta-miR-424-5p	39.278	16.148	−1.2823	5.21 × 10^−45^	down	**
bta-miR-361	23.528	9.6582	−1.2845	1.09 × 10^−27^	down	**
bta-miR-491	1.1250	0.4599	−1.2905	1.90 × 10^−2^	down	*
bta-miR-32	4.0598	1.6353	−1.3119	4.61 × 10^−6^	down	**
bta-miR-450b	1.2718	0.511	−1.3155	1.12 × 10^−2^	down	*
bta-miR-20a	15.212	6.0811	−1.3228	3.09 × 10^−19^	down	**
bta-miR-195	220.8	88.253	−1.323	1.65 × 10^−256^	down	**
bta-miR-19b	12.864	5.1102	−1.3319	1.17 × 10^−16^	down	**
bta-miR-205	186.85	72.871	−1.3585	2.62 × 10^−226^	down	**
bta-miR-450a	2.3968	0.9198	−1.3817	2.65 × 10^−4^	down	**
bta-miR-2285o	3.2283	1.2264	−1.3963	1.88 × 10^−5^	down	**
bta-miR-23a	2628.1	952.28	−1.4645	0.00	down	**
bta-miR-328	2.3479	0.8176	−1.5219	1.05 × 10^−4^	down	**
bta-miR-31	241.39	81.712	−1.5627	0.00	down	**
bta-miR-1388-3p	6.8968	2.1463	−1.6841	5.88 × 10^−13^	down	**
bta-miR-299	8.0707	2.4529	−1.7182	2.62 × 10^−15^	down	**
bta-miR-150	14.381	4.1903	−1.779	2.47 × 10^−27^	down	**
bta-miR-96	9.1958	2.6573	−1.791	3.56 × 10^−18^	down	**
bta-miR-331-5p	2.25	0.6132	−1.8755	1.10 × 10^−5^	down	**
bta-miR-136	1.9076	0.511	−1.9004	4.61 × 10^−5^	down	**
bta-miR-335	5.9185	1.5842	−1.9015	4.13 × 10^−13^	down	**
bta-miR-1247-5p	6.5055	1.7375	−1.9046	2.66 × 10^−14^	down	**
bta-miR-196b	13.305	3.2194	−2.0471	2.51 × 10^−30^	down	**
bta-miR-24	1.125	0.2555	−2.1385	8.30 × 10^−4^	down	**
bta-miR-23b-3p	1265.7	282.13	−2.1655	0.00	down	**
bta-miR-487b	16.386	3.5771	−2.1956	2.80 × 10^−40^	down	**
bta-miR-204	48.767	10.578	−2.2048	3.91 × 10^−117^	down	**
bta-miR-135a	1.5163	0.3066	−2.3061	4.30 × 10^−5^	down	**
bta-miR-655	1.8098	0.3577	−2.339	6.14 × 10^−6^	down	**
bta-miR-410	1.125	0.2044	−2.4605	2.79 × 10^−4^	down	**
bta-miR-26a	6964.3	1262.7	−2.4634	0.00	down	**
bta-miR-378b	2.0544	0.3577	−2.5219	4.60 × 10^−7^	down	**
bta-miR-380-3p	21.767	3.0661	−2.8276	2.55 × 10^−70^	down	**
bta-miR-145	3096.8	299.05	−3.3723	0.00	down	**

### 2.6. Verification of miRNA Expression by Real Time PCR

**Figure 4:**
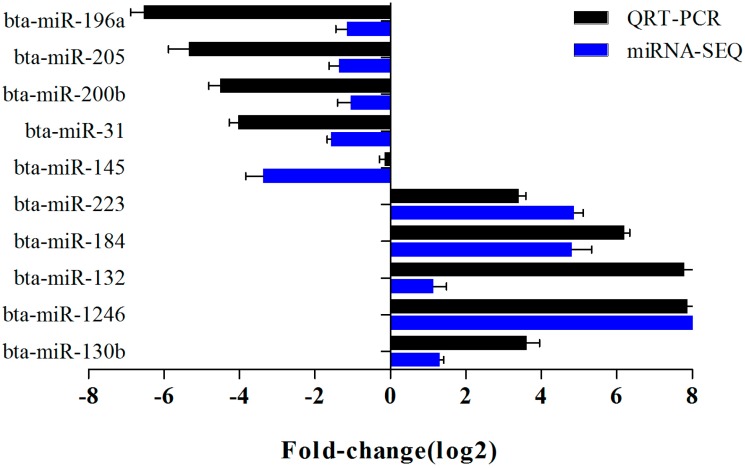
Comparison of the results of Solexa sequencing and qPCR.

The expression levels of 10 selected miRNAs were verified by real time PCR. The expression of miR-223, miR-184, miR-132, miR-1246 and miR-130b were up-regulated while miR-196a, miR-205, miR-200b, miR-31 and miR-145 were down-regulated, which was in agreement with the high-throughput sequencing results (Figure 4).

### 2.7. Target Gene Prediction

**Figure 5:**
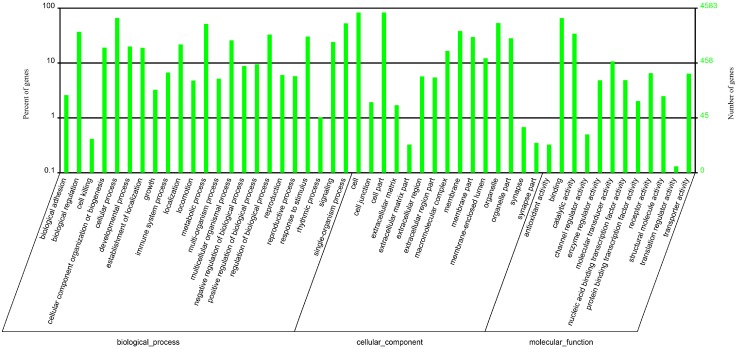
Gene ontology statistics.

After filtering out the differences in miRNA expression using RNA hybrid software (available online: http://bibiserv.techfak.uni-bielefeld.de/rnahybrid.), target gene predictions of the differentially expressed miRNA were performed using both strands of the affected miRNA genes. Of the 77 differentially expressed miRNAs between the *S. aureus* and control groups 19,792 target genes and 173,612 loci were predicted (Table 2). GO analysis and KEGG analysis of these target genes revealed that they were related with binding, catalytic activity, immune processes and microbial metabolism, endocytosis, and olfactory transduction signal pathways (Figure 5, Table 3).

**Table 2:** Partial list of predicted microRNA gene targets.

miRNA	Target Genes
miR-1246	*ATP2B4*, *MAP3K1*, *ADCK3*, *PSD2*, *SLC5A1*
miR-130b	*EXOC3L1*, *TIE1*, *BAZ2B*, *C3*, *GRAMD1C*
miR-145	*HSD3B7*, *SLCO4A1*, *PDIA4*, *ACADL*, *PTPN11*, *KRT9*, *RASSF6*, *RNF43*, *LAMC2*
miR-196a	*ADAP1*, *GPR97*, *POMT1*
miR-200b	*ARID3A*, *MLXIP*, *GPR110*
miR-205	*IL13RA2*, *COL5A2*, *ADM*, *CXCR2*, *XPO6*, *SPSB1*, *FMO5*, *PSMF1*
miR-31	*MEX3D*, *PFKFB3*, *ST3GAL3*, *IL2RB*, *ANKRD32*, *MGST1*
miR-184	*HSPA1L*, *SLC25A15*, *HEG1*, *MAPRE2*, *ACP6*, *SYNE2*
miR-223	*TMEM165*
miR-132	*IQCA1*

**Table 3:** Pathway annotation.

Pathway	Target Genes with Pathway Annotation (15970)	All Genes with This Type of Pathway Annotation (16078)	*p*-Value	*Q*-Value	Pathway ID
Olfactory transduction	1051 (6.58%)	1051 (6.54%)	0.0006579	0.2033013	Ko04740
Pathways in cancer	528 (3.31%)	528 (3.28%)	0.0268226	1.0000000	Ko05200
Focal adhesion	425 (2.66%)	425 (2.64%)	0.0548559	1.0000000	Ko04510

## 3. Discussion

Mastitis is a common disease in dairy cattle that causes serious economic losses to the dairy industry. To solve this problem it is particularly important to understand the immune responses related to mastitis at the molecular level. miRNAs have been proposed to participate in leukocyte reproduction, development, disease prevention and other activities through the regulation of target genes and have been used in research into immune regulation of bacterial infections. Currently, researchers have focused on understanding the roles of miRNA in immune regulation. miRNAs played a key role in regulating innate and adaptive immunity in humans and mice [17]. For example, miR-146a can modulate the innate immune response to bacterial infection by targeting the TNF receptor associated factor 6 (TRAF6) and interleukin receptor associated enzyme I (IRAK1) [18]. In recent years, miRNAs have also been increasingly used in the study of dairy cows [19,20,21]. Some key milk-related miRNAs were identified using a miRNA microarray assay of mammary gland tissue from dairy cows [22,23,24].

This study focused on the differences between two miRNA libraries derived from healthy breast tissue and breast tissue infected with *S. aureus* to provide new information for the in-depth study of the molecular mechanisms of mastitis. A total of 77 miRNA with significant expression differences were found between the *S. aureus* group and control group, with extremely significant differences associated with the expression of 74 miRNA.

Among these miRNA, miR-31 and miR-205 have been recognized as closely related to breast cancer [25] with relatively abundant expression levels in tumor tissues and relatively low expression in healthy tissues. In our study, the expression of miR-31 (log2 = −1.52) and miR-205 (log2 = −1.36) in breast mammary gland tissue infected with *S. aureus* appeared to be significantly down-regulated relative to the control, which is similar to the trend observed in breast cancer. A study indicated, in mammary cancer cell lines with a defective p53 signaling pathway, the overexpression of miR-31 inhibited proliferation and induced apoptosis; however, in wild-type cells, miR-31 had no effect [26]. Our data suggest that miR-31 could play a similar role in bovine mammary gland.

In our study, the expression of miR-223 in breast mammary tissue infected with *S. aureus* was significantly up-regulated relative to the control group (log2 = 4.88). miR-223 has been studied primarily in granulocytes and neutrophils, it negatively regulates the proliferation and differentiation of neutrophils through down-regulation of the transcription factor Mef2c, a known promoter of myeloid progenitor proliferation. In a miR-223 knockout mouse, increased neutrophil numbers, spontaneous inflammation in the lung and exaggerated tissue destruction was observed following LPS challenge. It supported a model in which miR-223 acts as a fine-tuner of granulocyte production and the inflammatory response [27]. miR-223 also has been identified as a specific and sensitive biomarker for sepsis in humans [28]. A previous study observed down-regulation of miR-223 in bovine blood monocytes challenged with *S. aureus* enterotoxin B [14], but in another study, miR-223 was upregulated in dairy cow mammary gland tissues infected with *streptococcus* [15]. Thus, a deeper analysis of the biological functions and effects of the expression of miR-223 in mammary epithelial cells is needed to clarify and it may participate in regulation of mastitis in dairy cows and could be used as a candidate for further research.

miR-132 has been shown to regulate the blood vessel growth and nerve degeneration and to play a role in controlling the inflammatory response. miR-132 expression increased after virus infection and regulated the immune response by targeting the inhibition of p300, which demonstrated an important role for miR-132 in the regulation of anti-viral immunity [29,30]. It was previously reported that the expression of miR-132 increased gradually over time after LPS-induced inflammation of rat alveolar macrophages, which suggested that miR-132 may be involved in regulation of the rat alveolar macrophages inflammatory response [31]. In this study, the expression of miR-132 in breast mammary gland tissue infected with *S. aureus* appeared to be significantly increased compared to the control, which indicated that miR-132 could also play a role in the regulation of immune responses tobacterial infections.

It is noteworthy that miR-1246 has the most significant difference (log2 = 8.41) in our study, yet there is very limited report on its function. Therefore one of our next steps will be focused on investigating its role in the inflammatory response of mastitis.

Biological analysis of miRNAs and their target genes in this study provided a useful approach for studying the immune regulation of mastitis. By comparing miRNA expression between the infected group and healthy controls, miRNAs with significant differences were screened as key factors to participate in the immune regulation of mastitis. Through miRNA target gene prediction, GO analysis and KEGG analysis, a “miRNAs—target genes—proteins” immune regulation network can be built as a foundation for further study.

## 4. Experimental Section

### 4.1. Animals

The three 2-year-old Holstein cows in mid-lactation used in this study were selected from a farm on the outskirts of Yangzhou, Jiangsu, China. The milk SCC <200,000 cells/mL, and cows do not have been treated for mastitis or any other disease. Milk samples were collected for bacteriology examination to confirm that none were sub-clinically infected with pathogen. Then cows were housed and fed individually, and had free access to water.

### 4.2. Construction of a Mastitis Model in Dairy Cows

The healthy Chinese Holstein cows were injected with a 5 mL suspension of 10^7^ CFU/mL *S. aureus* (ATCC29213, purchased from CGMCC) into the mammary glands using a tipless needle. The control group was injected with sterile PBS (Table S5).

### 4.3. Collection of Tissue Samples

After successful construction of the mastitis model was confirmed, mammary gland tissues were aseptically collected. In brief, surgeries were performed under aseptic conditions. Cows were sedated using intravenous administration of xylazine HCl. Cows were lying down and restrained to minimize movement. The biopsy site was carefully selected to avoid subcutaneous blood vessels as well as the cisternal region. The hair around the biopsy site was clipped and then washed and sterilized with iodine surgical scrub and 70% ethanol. For local anesthesia, 5 mL lidocaine HCl was administered subcutaneously. The biopsy site was then washed as described above three times. After washes, a 6–7 cm incision was made through the skin and underlying fascia to the point where the mammary gland capsule was visible. A small piece of mammary tissue was removed with a scalpel. Tissue (≥1 g) was blotted with sterile gauze to remove any visible milk secretions and connective tissue. Mammary tissue was then frozen immediately in liquid nitrogen. Once the tissue was collected, pressure was applied to the incision area until the cessation of bleeding. The skin incision was closed with surgical suture and iodine ointment was applied to the surgical site. The collected tissue samples were stored immediately in liquid nitrogen, delivered to the laboratory and stored at −70 °C.

### 4.4. Histologic Examination

The paraffin-fixed blocks were serially sectioned into 6–7 μm coronal slices and stored at −20 °C. For routine histological studies, paraffin sections were stained with hematoxylin and eosin. Hematoxylin-eosin stained sections were analyzed by light microscopy using a Nikon fluorescence microscope (Nikon, Tokyo, Japan).

### 4.5. RNA Sequencing

Total RNA was extracted from mammary gland tissues using the mirVana RNA Isolation Kit (Applied Biosystems, p/n AM1556, Carlsbad, CA, USA) and purified using the QIAGEN RNeasy^®^ Kit (QIAGEN, Mainz, Germany). The Agilent 2100 Bioanalyzer (Agilent Technologies, Palo Alto, CA, USA) was used for quality testing after purification. Total RNA samples were stored at −70 °C. A total of 5 μg of each RNA sample was sent to ShangHai Oebiotech Co. (Shanghai, China) for RNA sequencing. 

### 4.6. Data Analysis

After removing low-quality reads, adaptors, insufficient tags and sequences the length distributions of the clean reads were summarized and the common and specific tags between the two groups were analyzed.

rRNAs, tRNAs, snRNAs and other non-coding RNAs were identified and removed based on comparisons with the GenBank and Rfam databases. Known miRNAs in the samples were identified by comparison with the specified range in the miRNA database (miRBase, available online: http://www.mirbase.org) and repeated associated small RNAs (sRNAs) were identified by comparison with repeated sequences. Novel miRNAs and their secondary structures were predicted using Mireap from the unannotated sRNAs. Next, these known miRNAs were analyzed and evaluated through target prediction, GO enrichment analysis and KEGG pathway analysis for differential expression between the experimental and control groups.

### 4.7. Verification of miRNA Expression by qRT-PCR

The RNA was reverse transcribed to cDNA using the miRNA First-Strand cDNA Synthesis Kit (TIANGEN, Beijing, China). First, 2 μL 10× polymerase buffer, 4 μL 5× rATP Solution, 0.4 μL *E. coli* Poly (A) Polymerase (5 U/μL), and 2 μL total RNA (10 pg·μL^−1^~1 μg·μL^−1^) were added into ice-cold RNase-Free reaction tubes and supplemented with RNase-Free ddH2O to a total volume of 20 μL. After a brief centrifugation the mixture was incubated for 60 min at 37 °C. Second, 2 μL Poly (A) reaction mixture obtained in the first step was added to a solution containing 10× RT Primer, 2 μL 10× RT Buffer, 1 μL Super Pure dNTPs, 1 μL RNasin (40 U/μL), 0.5 μL Quant RTase, and 11.5 μL RNase-Free ddH_2_O. After a brief centrifugation, the reaction solution was incubated for 60 min at 37 °C prior tocDNA synthesis.

The expression levels of mature miRNA were detected using the SYBR green method. Both miRNA-specific primers and universal primers were used (the miRNA-specific primers were synthesized by Shanghai Biological Technology Co. and the universal primers were provided by the miRcute miRNA qPCR Detection kit, TIANGEN). Bovine 18S rRNA was used as a miRNA control. Real-time PCR was performed on an ABI7300 system (Applied Biosystems) using the miRcute miRNA qPCR Detection kit (SYBR Green) (TIANGEN). The PCR was performed in a reaction system with 10 μL 2× miRcute miRNA Premix (containing SYBR with ROX), 0.4 μL specific upstream primer, 0.4 μL universal primer, 1.6 μL 50× ROX Reference Dye and 2 μL cDNA; the reaction tubes were supplemented with RNase-Free ddH2O to a total volume of 20 μL. The reaction mixtures were incubated in a 96-well plate at 95 °C for 2 min, followed by 40 cycles of 94 °C for 20 s and 60 °C for 34 s. All reactions were run in triplicate. Fold changes were determined by the threshold cycle (*C*_t_). Fold changes of miRNA expression were calculated using the 2^ΔΔ*C*t^ method [32]. Primers designed for real-time PCR are listed in (Table 4).

**Table 4:** Primer sequences for real-time PCR.

miRNA	Primer Sequences(5'–3')
bta-miR-223	CCTGTCAGTTTGTCAAATACCCCA
bta-miR-31	GGAAGGCAAGATGCTGGCA
bta-miR-205	TCCTTCATTCCACCGGAGTCTG
bta-miR-145	GTCCAGTTTTCCCAGGAATCCCT
bta-miR-132	TAACAGTCTACAGCCATGGTCGAAA
bta-miR-130b	AGCAGGCAGTGCAATGATGA
bta-miR-200b	GCTGACGGTGCTAATACTGCCT
bta-miR-1246	GAATGGATTTTTGGAGCAGGAA
bta-miR-196a	GCTGCGACCGTAGGTAGTTTCAT
bta-miR-184	TGGACGGAGAACTGATAAGGGTAAA
bta-S18(F)	CACCGAGGATGAGGTGGA
bta-S18(R)	TATTGGCGTGGATTCTGC

## 5. Conclusions

In our study, we characterized the miRNAs of bovine mammary glands challenged with *S. aureus* bacteria and detected 370 known bovine miRNAs and 341 novel miRNAs. GO analysis showed significant enrichments of predicted target genes of differentially regulated miRNAs in different functional groups such as cells, binding, catalytic activity and immune processes *etc.* and KEGG pathways of the diverse environments, endocytosis, olfactory transduction, pathways involved in cancer. Our study provides an experimental basis to reveal the cause and regulatory mechanism of mastitis and the potential role for miRNAs as biomarkers in early diagnosis of mastitis and in development of control measures.

## Supplementary Materials

Supplementary materials can be found at http://www.mdpi.com/1422-0067/16/03/4997/s1.

## Abbreviations

RB: right back
LF: left front
LB: left back
