# Supplementary material for: Transcriptome MicroRNA Profiling of Bovine Mammary Glands Infected with Staphylococcus aureus
Source: Int J Mol Sci. 2015 Mar 4;16(3):4997–5013. doi: 10.3390/ijms16034997 (PMC4394461; doi:10.3390/ijms16034997)
Supplement: Supplementary file 1 [file ijms-16-04997-s001.pdf]

Supplementary Information

Table S1. Summary of known miRNA in each sample.

	miRNA	miRNA *	miRNA-5p	MiRNA-3p	miRNA Precursors	Unique sRNAs Matched to miRNA Precursors	Total sRNAs Matched to miRNA Precursors
Known miRNA in miRbase	620	0	82	81	798		
Control group	358	0	53	51	488	8066	14,380,650
S. aureus group	370	0	55	53	511	8539	12,890,726

Table S2. Distribution of small RNA among different categories in control group.

Category	Unique sRNAs	Percent (%)	Total sRNAs	Percent (%)
Total	466,207	100%	20,444,199	100%
miRNA	7957	1.71%	14,373,694	70.31%
rRNA	27,108	5.81%	99,431	0.49%
snRNA	1253	0.27%	2475	0.01%
snoRNA	2772	0.59%	19,791	0.1%
tRNA	10,378	2.23%	59,953	0.29%
unann	416,739	89.39%	5,888,855	28.8%

Table S3. Distribution of small RNA among different categories in *S. Aureus* group.

Category	Unique sRNAs	Percent (%)	Total sRNAs	Percent (%)
Total	882,795	100%	19,568,797	100%
miRNA	8367	0.95%	12,883,504	65.84%
rRNA	70,071	7.94%	725,171	3.71%
snRNA	6547	0.74%	211,311	1.08%
snoRNA	4733	0.54%	24,778	0.13%
tRNA	14,199	1.61%	73,380	0.37%
unann	778,878	88.23%	5,650,653	28.88%

Table S4. Summary of small RNA sequencing data.

	Control Group		*S. aureus* Group	
Type	Count	Percent (%)	Count	Percent (%)
total_reads	21,293,853		18,588,177	
high_quality	21,253,265	100%	18,565,803	100%
3' adapter_null	5959	0.03%	3756	0.02%
insert_null	8499	0.04%	3612	0.02%
5' adapter_contaminants	330,968	1.56%	22,295	0.12%
smaller_than_18nt	60,374	0.28%	31,238	0.17%
polyA	465	0.00%	127	0.00%
clean_reads	20,847,000	98.09	18,504,775	99.67%

Table S5. Injection conditions.

Group	Injection Sites		
S. aureus	1RB	2RB	3LB
Control	1LF	2LF	3LF
